# Unraveling the insomnia puzzle: sleep reactivity, attention deficit hyperactivity symptoms, and insomnia severity in ADHD Patients

**DOI:** 10.3389/fpsyt.2024.1528979

**Published:** 2025-01-22

**Authors:** Hilal Uygur

**Affiliations:** Department of Psychiatry, Erzurum Faculty of Medicine, University of Health Sciences, Erzurum, Türkiye

**Keywords:** attention deficit hyperactivity, ford insomnia response to stress test, insomnia, sleep, sleep reactivity

## Abstract

**Introduction:**

Insomnia is the most common sleep problem among adults with attention deficit hyperactivity disorder (ADHD). The severity of insomnia can exacerbate the symptoms of ADHD. Identifying the predictive factors that contribute to insomnia severity may be crucial in treating insomnia among adults with ADHD. One potential factor contributing to insomnia is sleep reactivity, which refers to the tendency to experience insomnia triggered by stress. This phenomenon, also known as vulnerability to stress-related insomnia, reflects the extent to which individuals are prone to developing insomnia in response to stressful situations. To date, sleep reactivity in adults with ADHD has not been studied. This study aimed to investigate the relationship between sleep reactivity, attention deficit hyperactivity symptoms, and the severity of insomnia in adults with ADHD.

**Methods:**

This study included 61 adults with ADHD with no comorbid psychiatric or medical diseases and 61 adult healthy controls (HCs) matched for age, sex, body mass index, and years of education. All participants completed the sociodemographic data form, Ford Insomnia Response to Stress Test (FIRST), Pittsburgh Sleep Quality Index (PSQI), Insomnia Severity Index (ISI), Adult ADHD Self-Report Scale (ASRS), and Wender Utah Rating Scale (WURS).

**Results:**

Sleep reactivity, insomnia severity, and poor sleep quality were higher in adults with ADHD compared to HCs. Higher sleep reactivity was associated with worse sleep outcomes in both groups, whereas higher sleep reactivity was associated with severe ADHD symptoms in the HCs group. In the ADHD group, insomnia severity was predicted by higher sleep reactivity, ASRS, and being female (R^2^ = 0.32, F (6, 61) = 4.36, p = 0.001), not age, ADHD medication use, and WURS.

**Conclusion:**

Our findings suggest that higher sleep reactivity, increased ADHD symptoms, and being female may predict the severity of insomnia in adults with ADHD.

## 1 Introduction

Attention deficit hyperactivity disorder (ADHD) is a neurodevelopmental disorder characterized by inattention, impulsivity, and/or hyperactivity (1). The prevalence of ADHD in adults is reported to be 2.5% in epidemiological studies and 2.8% in the most recent World Health Organization’s Mental Health Survey conducted across 10 countries (2, 3). It is important to note that the incidence of ADHD in adults is likely underestimated, as ADHD is often perceived as a childhood disorder that improves with age, and the criteria in the Diagnostic and Statistical Manual of Mental Disorders (DSM) are not adequately sensitive to the adult population. Therefore, adults with ADHD require greater attention (4). ADHD can significantly impair various aspects of daily life, including interpersonal relationships, academic performance, occupational success, and the ability to manage everyday tasks (5). In addition to its core symptoms, adults with ADHD often develop comorbid psychiatric disorders, such as anxiety, mood disorders, and substance use disorders (6).

Sleep disturbances are common and have significant clinical implications for adults with ADHD. Approximately 40–70% of adults with ADHD experience symptoms of insomnia, which is significantly higher than the rate reported in the general population (7). Insomnia can adversely affect cognitive functions such as attention, learning, memory, and executive function in ADHD. A bidirectional relationship is present between insomnia and ADHD symptoms: while more severe ADHD symptoms can exacerbate insomnia, increased severity of insomnia can also worsen ADHD symptoms (8, 9). Similarly, a comparable relationship occurs between other psychiatric disorders and insomnia, where psychiatric comorbidities can elevate the risk of insomnia, and conversely, insomnia can intensify the severity of psychiatric comorbidities (8, 10). Therefore, treating insomnia may not only improve ADHD symptoms but also alleviate comorbid mental health issues in adults with ADHD. A recent longitudinal study confirmed that adults with ADHD whose insomnia symptoms remitted showed improvements in both ADHD symptoms and their overall quality of life (5). The management of insomnia in adults with ADHD has a significant challenge due to the lack of ideal pharmacological treatments and the limited access to cognitive behavioral therapy-insomnia (CBT-I), which is considered the gold standard for insomnia treatment (11, 12). Additionally, ADHD medications and their various formulations (e.g., stimulants and non-stimulants, short-acting and long-acting) can have significant and varying effects on sleep in adults with ADHD. Delayed sleep onset or insomnia may increase when stimulants are first introduced, although daytime sleepiness is associated with non-stimulants (13). Consequently, identifying factors that predispose insomnia is crucial for developing effective preventive treatments.

Stress-diathesis models are widely accepted as etiological frameworks for a variety of psychiatric disorders, including depression, post-traumatic stress disorder, schizophrenia, and insomnia (14). According to these models, exposure to stress increases the likelihood of insomnia, though not everyone exposed to stress develops insomnia. This suggests that individual differences in stress regulation are crucial in determining whether insomnia will develop. Sleep reactivity refers to the extent to which stressors disrupt sleep, and it is a measure of an individual’s sensitivity to stress in relation to their sleep patterns (15, 16). Other terms used to describe sleep reactivity include “sleep system sensitivity,” “stress reactivity of the sleep system,” and “vulnerability to insomnia” (17, 18). Sleep reactivity is a normal phenomenon; all individuals experience difficulty sleeping when faced with specific stressors. Even minor challenges, such as unfamiliar sleeping environments or slight circadian shifts, can lead to transient sleep difficulties for many. However, individuals with higher sleep reactivity are more vulnerable to insomnia, even after the initial stressor has resolved, whereas those with lower sleep reactivity experience mild sleep disruptions that return to normal without serious complications (19, 20). The Ford Insomnia Response to Stress Test (FIRST) is a self-rated questionnaire that effectively measures sleep reactivity and has been shown to predict both the onset and persistence of insomnia symptoms over time (21–23). Additionally, one study found that higher sleep reactivity and insomnia can synergistically aggravate depressive symptoms (24). Therefore, interventions targeting sleep reactivity may also be beneficial in the treatment of mental health symptoms.

Research on the link between ADHD and stress has primarily focused on both physiological stress responses and the impact of daily stressors. Generally, research shows that adults with ADHD exhibit higher physiological stress response, as measured by salivary cortisol levels, and report greater subjective stress compared to non-diagnosed adults (25–27). Specifically, stress levels in adults with ADHD are typically higher both when a stressor is present or when it is anticipated. Additionally, one study found that adults with ADHD have more difficulty recovering from stress (26). These findings suggest that adults with ADHD may be more susceptible to experiencing elevated stress. Moreover, research indicates that adults with ADHD exhibit diminished coping abilities in the face of stress which likely exacerbates the impact of stressors (28, 29). Given the high stress levels and poor stress management in adults with ADHD compared to those without, sleep reactivity may be higher in this population. Therefore, sleep reactivity may be a predisposing factor for insomnia in adults with ADHD and may worsen ADHD symptoms by increasing the severity of insomnia.

Identifying adults with ADHD who have higher sleep reactivity and interventions to reduce sleep reactivity may help prevent insomnia. As a result, the worsening of ADHD and depressive symptoms can be prevented. To date, the role of sleep reactivity in adults with ADHD has not been examined. In the present study, we collected cross-sectional self-report data on sleep reactivity, insomnia severity, sleep quality, and ADHD symptoms (childhood and current ADHD symptoms) from 61 adults with ADHD no comorbid psychiatric or medical disease and 61 adult healthy controls (HCs) matched for age, gender, body mass index (BMI), and education level. We hypothesized that adults with ADHD compared to HCs would report higher sleep reactivity, more severe insomnia, and poor sleep quality. Additionally, we hypothesized that individuals with high sleep reactivity, whether in the ADHD or HC group, would experience worse sleep quality, more severe insomnia, and higher ADHD symptom severity compared to those with low sleep reactivity. Finally, we aimed to identify predictive factors of insomnia severity in adults with ADHD through regression analysis, focusing on sleep reactivity, age, gender, ADHD symptoms, and ADHD medication use.

## 2 Materials and methods

### 2.1 Participants and procedure

Since this study is the first to investigate sleep reactivity in adults with ADHD, there were no previous studies directly addressing the required sample size for this type of research. Therefore, sample size estimation was based on estimates based on similar research. Based on assumptions from similar studies, we determined that a sample size of a minimum of 50 adults with ADHD and 50 HCs would be sufficient to achieve reliable and valid results.

The study included 61 adults diagnosed with ADHD and 61 HCs. The adults with ADHD in this study were consecutively selected from those attending the psychiatry outpatient clinic. All patients were diagnosed by a licensed psychiatrist. The inclusion criteria for the patient group were: 1) a diagnosis of ADHD according to the 5th edition of the DSM; and 2) voluntary participation in the study. The exclusion criteria included: 1) a comorbid mental illness (including insomnia disorder); 2) use of any medication except ADHD medications; 3) smoking, alcohol consumption, or substance use; 4) chronic disease; 5) a mental deficit preventing the completion of the scales. For the HCs, the inclusion criteria were: 1) no history of mental illness, either past or present; and 2) voluntary participation in the study. Exclusion criteria for HCs included: 1) use of any medication; 2) chronic disease; 3) smoking, alcohol consumption, or substance use; 4) a mental deficit preventing the completion of the scales. HCs were randomly selected from hospital staff and matched with the patient group based on age, sex, BMI, and educational level. In order to recruit HCs, an announcement regarding the study, including the conditions of participation, was made throughout the hospital. They were invited to participate if they expressed interest in the study. This was intended to ensure random and unbiased participation.

The study adhered to the principles of the Declaration of Helsinki. Informed consent was obtained from all participants before their inclusion in the study. Ethical approval was issued by the Scientific Research Ethics Committee of Erzurum Faculty of Medicine, University of Health Sciences (approval number: 2024/02-38). The research was conducted between March 1, 2024, and August 1, 2024.

All participants completed a socio-demographic questionnaire, which included questions on age, sex, weight, height, and educational level. Additionally, all subjects responded to several scales: the FIRST, the Insomnia Severity Index (ISI), the Pittsburgh Sleep Quality Index (PSQI), the Wender Utah Rating Scale (WURS), and the Adult ADHD Self-Report Scale (ASRS). Sleep parameters were performed with the most commonly used self-psychometric scales in the literature and showed high reliability in our study.

### 2.2 Measures

#### 2.2.1 Ford insomnia response to stress test

Sleep reactivity was assessed using the FIRST (21). It is a self-report questionnaire to assess the relationship between stress and insomnia. The FIRST-T is a self-administered questionnaire that evaluates the likelihood of insomnia about nine specific stress-inducing events (e.g., “after a stressful experience during the day” and “before an important meeting the next day”). Each item is rated on a scale from 1 to 4, and the total score ranges from 9 to 36 points, with higher scores indicating greater levels of sleep reactivity (21). The FIRST has demonstrated adequate psychometric properties. The Turkish version of the FIRST was validated by Uygur et al. (30). In our study, we found adequate internal consistency for the FIRST (Cronbach’s α = 0.78, McDonald’s ω = 0.79). It has been suggested that median values of the FIRST can serve as cut-off points for distinguishing between high and low sleep reactivity (17, 30). Our study found a median FIRST score of 22 for ADHD patients and 17 for HCs, which we accepted as cut-off values to differentiate between high and low sleep reactivity in both groups.

#### 2.2.2 Insomnia severity index

The ISI was used to evaluate the severity of insomnia. The ISI consists of seven items that assess various aspects of insomnia, including difficulties with sleep onset and maintenance, dissatisfaction with sleep quality, and the impact of sleep problems on daily functioning (31). The Turkish version of the ISI also consists of 7 items, each rated on a scale from 0 to 4, resulting in a total score that ranges from 0 to 28. A total score of 0-7 indicates no insomnia, while a score of 15 or higher is considered indicative of clinical insomnia. The Turkish version of the ISI was validated by Boysan et al. and has demonstrated adequate validity (32). In our study, we found robust internal consistency for the ISI (Cronbach’s α = 0.80, McDonald’s ω = 0.82). We accepted 15 points and above as the cut-off value for clinical insomnia in our study.

#### 2.2.3 Pittsburg sleep quality index

The PSQI is a 19-item self-report questionnaire that assesses sleep quality and its disturbances during the last month. The PSQI is scored from 0 to 21, with higher scores indicating greater sleep disturbances and poor sleep quality (33). Agargün et al. conducted the psychometric properties Turkish version of the PSQI and demonstrated adequate validity and reliability (34). In our study, we found adequate internal consistency for PSQI (Cronbach’s α = 0.74, McDonald’s ω = 0.75).

#### 2.2.4 Adult ADHD self-report scale

The clinical severity of ADHD was evaluated with the ASRS (35). It is an 18-item self-report questionnaire to assess ADHD symptoms in adults. Each item is rated from 0 to 4, and the total ASRS score ranges between 0 and 72. Higher scores indicate more severe ADHD symptoms. Evren et al. adapted and validated the Turkish version (36). In our study, we found robust internal consistency for ASRS (Cronbach’s α = 0.84, McDonald’s ω = 0.86).

#### 2.2.5 Wender utah rating scale

The clinical severity of ADHD during childhood was evaluated with the WURS. It is a 25-item self-report tool to assess retrospectively the severity of childhood ADHD symptoms in adults. The WURS has demonstrated strong psychometric properties (37). Each item is rated from 0 to 4, and the total WURS score varies between 0 and 100. Higher scores indicate more severe childhood ADHD symptoms. The Turkish adaptation of the WURS was validated in a study by Öncü et al. (38). In our study, we found adequate internal consistency for WURS (Cronbach’s α = 0.78, McDonald’s ω = 0.79).

### 2.3 Statistical analysis

Statistical analyses were performed using SPSS 23.0 and Jamovi 2.3.28. Descriptive statistics were presented as numbers, percentages, means, and standard deviations. Data normality was assessed based on skewness and kurtosis values. For normally distributed data, the Student’s t-test was used for comparisons, while the Mann-Whitney U test was used for non-normally distributed data. The Chi-square test was used to compare categorical variables. For correlation analyses, Pearson correlation was used for normally distributed variables, and Spearman correlation was used for non-normally distributed variables.

We compared sleep reactivity, insomnia severity, sleep quality, and ADHD symptoms between the ADHD and HCs groups. Both groups were divided into high and low sleep reactivity subgroups. Sleep scales and ADHD symptoms were compared between these high and low sleep reactivity groups. Correlation analyses were conducted between the scales in both the ADHD and HC groups. Additionally, we performed linear regression analysis to identify factors predicting insomnia severity in ADHD patients. Statistical significance was set at p < 0.05.

## 3 Results

### 3.1 Participants characteristics and scales about sleep and ADHD symptoms

The study sample consisted of 61 adults with ADHD and 61 HCs. Twenty-four patients in the ADHD group were female; the mean age of the ADHD group was 25.3 (SD 6.4) years. Twenty-one patients in the HCs group were female; the mean age of HCs was 26.1 (SD 3.6) years. There was no significant difference between the two groups in terms of age, sex, BMI, and years of education. 54.1% (n = 33) of adults with ADHD used ADHD medication (stimulants or atomoxetine). Adults were usually diagnosed with ADHD in childhood and some of them were receiving ADHD treatment while others were not.

The mean scores of the FIRST, ISI, PSQI, ASRS, and WURS in the ADHD group were, respectively, 22.8 (SD 5.9), 11.2 (SD 6.5), 8.8 (SD 3.8), 43.4 (SD 11.6), and 44.8 (SD 17.9). The mean scores of the FIRST, ISI, PSQI, ASRS, and WURS in the HCs group were, respectively, 22.8 (SD 5.9), 11.2 (SD 6.5), 8.8 (SD 3.8), 43.4 (SD 11.6), and 44.8 (SD 17.9). Significantly, all scale scores were higher in the ADHD group. In the ADHD group, 16% had clinical insomnia according to ISI, and 85.2% were poor sleepers according to PSQI. On the other hand, 4.9% had clinical insomnia according to ISI, and 62.3% were poor sleepers according to PSQI. **Table 1** presents the participants’ characteristics and scales as a comparison of the two groups.

**Table 1:** Demographic and sleep variables in ADHD and HCs group.

Variables	ADHD group (n = 61)	HCs group (n = 61)	*p values*
**Age, years**	25.32 ± 6.45	26.18 ± 3.68	0.372^b^
**Sex, female/male (n)**	24/37	21/40	0.573^a^
**BMI, kg/m^2^**	25.03 ± 4.93	24.45 ± 3.53	0.452^b^
**Education years**	16.55 ± 2.14	16.72 ± 1.31	0.612^b^
**FIRST**	22.83 ± 5.94	18.14 ± 4.88	**< 0.001**^**b**^
**ISI**	11.29 ± 6.55	6.57 ± 3.77	**< 0.001**^**b**^
**PSQI**	8.85 ± 3.84	5.77 ± 3.13	**< 0.001**^**b**^
**ASRS**	43.44 ± 11.67	18.80 ± 10.32	**< 0.001**^**b**^
**WURS**	44.80 ± 17.98	13.13 ± 9.86	**< 0.001**^**b**^
**Clinical Insomnia (%)**	26.2	4.9	**< 0.001**^**b**^
**Poor Sleeper (%)**	85.2	62.3	**< 0.001**^**b**^

a chi-square.

b Student t test.

Data are reported as mean ± standard deviation-SD and number. BMI, Body Mass Index; FIRST, Ford Insomnia Response to Stress Test; ISI, Insomnia Severity Index; PSQI, Pittsburg Sleep Quality Index; ASRS, Adult ADHD Self-Report Scale; WURS, Wender Utah Rating Scale.

Bold numbers/values: Statistically significant.

### 3.2 Comparisons of scales in high-low sleep reactivity groups

As in previous studies, we accepted the median score of the FIRST-T as the cut-off value for discriminating between high and low sleep reactivity. We divided both adults with ADHD and HCs into two groups: a low FIRST-T score group and a high FIRST-T score group. We found the cutoff score for high sleep reactivity is 22 in the ADHD group, and is 17 in the HCs group.

We conducted a comparison between the high and low sleep reactivity groups in both the ADHD and HCs groups. In the ADHD group, there was no significant difference between those with high sleep reactivity and those with low sleep reactivity in terms of age, sex, ASRS, and WURS, while FIRST, ISI, and PSQI were significantly higher in the high sleep reactivity group. In the HCs group, the high sleep reactivity group had a higher proportion of females, and their mean age was lower than that of the low sleep reactivity group. All scales were significantly higher in the high sleep reactivity group than in the low sleep reactivity group. **Table 2** presents the comparisons of high and low sleep reactivity in the ADHD and HC groups.

**Table 2:** Comparisons of demographic and sleep variables in high-low sleep reactivity groups both ADHD and HCs groups.

Variables	Groups	High-FIRST group	Low-FIRST group	*p values*
**Age**	ADHD group	26.06 ± 7.17	24.61 ± 5.70	0.38^b^
	HCs group	25.03 ± 2.48	27.29 ± 4.30	**0.02**^**b**^
**Sex**	ADHD group	13/17 (f/m)	11/20 (f/m)	0.53^a^
	HCs group	16/14 (f/m)	5/26 (f/m)	**0.02**^**b**^
**FIRST**	ADHD group	27.93 ± 3.11	17.90 ± 3.17	**< 0.001**^**b**^
	HCs group	22.30 ± 3.04	14.12 ± 2.20	**< 0.001**^**b**^
**ISI**	ADHD group	13.23 ± 6.18	9.41 ± 6.45	**0.02**^**b**^
	HCs group	8.33 ± 3.94	4.87 ± 2.72	**< 0.001**^**b**^
**PSQI**	ADHD group	10.03 ± 3.46	7.70 ± 3.89	**0.01b**
	HCs group	7.40 ± 3.12	4.19 ± 2.22	**< 0.001**^**b**^
**ASRS**	ADHD group	44.46 ± 11.30	42.45 ± 12.12	0.50^b^
	HCs group	23.40 ± 9.20	14.35 ± 9.47	**< 0.001**^**b**^
**WURS**	ADHD group	46.16 ± 18.29	43.48 ± 17.87	0.56^b^
	HCs group	16.60 ± 9.92	9.77 ± 8.70	**0.006**^**b**^

a chi-square.

b Student t test.

Data are reported as mean ± standard deviation-SD and number. FIRST, Ford Insomnia Response to Stress Test; ISI, Insomnia Severity Index; PSQI, Pittsburg Sleep Quality Index; ASRS, Adult ADHD Self-Report Scale; WURS, Wender Utah Rating Scale. Cut off values of FIRST ≥ 22 for ADHD group, FIRST ≥ 17 for HCs group.

Bold numbers/values: Statistically significant.

### 3.3 Correlations of scales in ADHD and HCs groups

We examined the correlation between the scales in both ADHD and HCs groups. In the ADHD group, significant correlations were found between FIRST and ISI and PSQI, while no significant correlations were found between FIRST and ASRS and WURS. However, in the HCs group, significant correlations were found between FIRST and ISI, PSQI, ASRS and WURS. **Table 3** presents the correlations of scales in ADHD and HCs groups.

**Table 3:** Correlations of scales in ADHD and HCs groups.

Scales	Groups	FIRST	ISI	PSQI	ASRS	WURS
**FIRST**	ADHD group	–				
	HCs group	–				
**ISI**	ADHD group	0.365**	–			
	HCs group	0.483**	–			
**PSQI**	ADHD group	0.371**	0.808**	–		
	HCs group	0.474**	0.657**	–		
**ASRS**	ADHD group	0.173	0.343**	0.247	–	
	HCs group	0.504**	0.385**	0.420**	–	
**WURS**	ADHD group	0.165	0.273*	0.216	0.278*	–
	HCs group	0.344**	0.305*	0.419**	0.624**	–

Pearson correlation test used. Data are reported as correlation coefficients. FIRST, Ford Insomnia Response to Stress Test; ISI, Insomnia Severity Index; PSQI, Pittsburg Sleep Quality Index; ASRS, Adult ADHD Self-Report Scale; WURS, Wender Utah Rating Scale.

*p< 0.05, **p <0.01

### 3.4 Predictors of insomnia severity in ADHD patients

As a result of the regression analysis was significant (F (6, 61) = 4.36, p = 0.001) and the independent variables explained 32% of the change in insomnia severity. According to the results of this analysis, FIRST, ASRS and being female were found to be significant in the model, while age, ADHD medication use, and WURS were found to be insignificant. **Table 4** presents the factors predicted insomnia severity in ADHD group.

**Table 4:** The factors predicted insomnia severity in ADHD group.

Predictor		Estimate		SE	t	p
**Intercept**		-10.943		5.182	-2.11	0.039
**FIRST**		0.344		0.134	2.56	0.013
**ASRS**		0.206		0.067	3.06	0.003
**WURS**		0.038		0.047	0.81	0.421
**Age**		0.031		0.122	0.25	0.797
**Sex:**						
** Male – Female**		4.243		1.611	2.63	0.011
**ADHD_Drug:**						
** no – yes**		0.641		1.593	0.40	0.689

Model 1	R	R²	F	df1	df2	p
	0.571	0.327	4.36	6	54	0.001

Linear - regression analyse. FIRST, Ford Insomnia Response to Stress Test; ISI, Insomnia Severity Index; ASRS, Adult ADHD Self-Report Scale; WURS, Wender Utah Rating Scale.

## 4 Discussion

In this study, the aim was to explore the role of sleep reactivity in the insomnia puzzle in adults with ADHD. Firstly, we compared sleep reactivity, insomnia severity, sleep quality, and ADHD symptoms between adults with ADHD and HCs. Second, we compared high and low sleep reactivity groups, which included both ADHD and HC groups. Finally, we analyzed the factors that predict insomnia severity in adults with ADHD.

In our study, the first key finding was that 26.2% of adults with ADHD had clinical insomnia, and 85.2% reported poor sleep quality. Between 43% and 85% of adults with ADHD experience insomnia symptoms, a prevalence significantly higher than that observed in the general population (39, 40). Consistent with previous studies, we found that adults with ADHD experienced more severe insomnia and poor sleep quality compared to HCs (39–42).

The second key finding was that adults with ADHD reported higher levels of sleep reactivity compared to HCs. This finding extends previous knowledge about sleep reactivity in adults with ADHD and emphasizes the pathogenicity of sleep reactivity in adults with ADHD. Several studies suggest that ADHD symptoms are positively correlated with perceived stress, and ADHD patients tend to have lower problem-solving skills (22, 43). As a result, adults with ADHD may be more likely to experience stress during minor life events, which could lead to increased sleep reactivity, thereby enhancing the likelihood of stress-induced insomnia.

The third key finding of the study was that adults with ADHD with high sleep reactivity reported greater severity of insomnia and poorer sleep quality. However, there was no significant difference in ADHD symptoms between patients with high and low sleep reactivity. Correlation analysis supported these findings, showing a significant positive correlation between sleep reactivity and both insomnia severity and poor sleep quality in adults with ADHD, but no correlation with ADHD symptoms. One possible explanation for the lack of difference in ADHD symptoms and the absence of a correlation is the use of ADHD medications by some patients.

It is important to note that high insomnia severity in adults with ADHD can lead to the onset of other mental health disorders, such as mood disorders and substance use. Therefore, it is crucial to identify adults with ADHD with high sleep reactivity to prevent the development and exacerbation of insomnia (42). Previous research has also explored sleep reactivity in cancer patients and pregnant women. Findings suggest that high sleep reactivity in cancer patients was a predictor of insomnia and was associated with increased levels of depression, anxiety, and suicide risk in pregnant women (22, 43).

The fourth key finding was that HCs with high sleep reactivity reported higher levels of insomnia, poor sleep quality, and more severe symptoms of ADHD (both current and childhood). Consistent with these findings, in the correlation analysis, sleep reactivity showed a significant positive correlation with insomnia severity, poor sleep quality, and ADHD symptoms. This is the first study in the literature to demonstrate a correlation between sleep reactivity and ADHD symptoms in HCs. It is known that sleep deprivation significantly impairs cognitive functions. High sleep reactivity may have negatively affected attention processes as a result of severe sleep deprivation, or individuals with high ADHD symptoms may be more sensitive to stressful events and, therefore, leading to higher sleep reactivity (29, 44, 45).

The fifth key finding revealed that there were no significant differences in age and gender between high and low sleep reactivity groups in adults with ADHD. However, among HCs, individuals with high sleep reactivity were more likely to be female and younger. Numerous studies have identified being female as a risk factor for insomnia, and females tend to be more sensitive to stress (46–48). Therefore, we anticipate higher levels of sleep reactivity in females.

The sixth key finding indicated that ADHD treatment did not predict the severity of insomnia. In our study, we included both adults with ADHD who were receiving medication and those who were not. Some patients were on stable medication regimens, while others had no current treatment. As such, the potential impact of ADHD medications on sleep was an important consideration. However, our findings did not reveal a significant relationship between ADHD treatment and insomnia severity. Previous studies have shown that the relationship between ADHD medications and sleep outcomes is complex, with some reporting improvements in sleep quality with certain medications, while others indicate potential negative effects such as insomnia or daytime sleepiness (13, 49). These conflicting results may be due to factors such as medication type, dosage, and individual differences, which may explain why we did not observe a clear link between ADHD treatment and sleep disturbances in our sample.

The final and most significant finding of our study was that severe insomnia in adults with ADHD was predicted by higher sleep reactivity, female gender, and greater ADHD symptom severity. While being female and the severity of ADHD symptoms have previously been identified as risk factors for insomnia in adults with ADHD (50). Our study is the first to show that sleep reactivity predicts insomnia severity in this population. Adults with ADHD may experience a higher incidence of insomnia due to this factor. The FIRST self-report scale can accurately predict high sleep reactivity in adults with ADHD, and this simple, rapid test could help prevent the development of insomnia in these patients, potentially averting severe insomnia. The increased severity of insomnia in adults with ADHD can negatively impact clinical outcomes (41, 42), exacerbating symptoms such as inattention and impulsivity. Insomnia treatment may reduce ADHD symptoms in these patients, but accessing CBT-I can be challenging. Furthermore, pharmacological treatment for insomnia may worsen ADHD symptoms or cause psychological and physiological dependence (51). Therefore, early identification of insomnia predictors and timely intervention could be a more cost-effective approach to managing these comorbid conditions.

### 4.1 Limitations

It was important to acknowledge the limitations of this study. First, the study is cross-sectional. Therefore, we cannot establish directionality or causality among sleep reactivity, insomnia severity, and ADHD symptoms. Second, the sample size is relatively small. However, considering that we used strict exclusion criteria such as comorbid mental illnesses, smoking, alcohol, and substance use, this sample size is quite sufficient. Third, we did not utilize objective sleep measurements, such as polysomnography or actigraphy. While these measurements would strengthen results but cost more. Despite these limitations, this study also has several important strengths. To our knowledge, this is the first study to investigate the relationship between sleep reactivity, ADHD symptoms, and insomnia severity in adults with ADHD. Additionally, we used an optimal set of validated instruments (FIRST, ISI, PSQI, ASRS, and WURS) in this study. Furthermore, the rigorous exclusion and inclusion criteria helped minimize the impact of potential confounding factors.

## 5 Conclusion

Early identification of adults with ADHD who are at heightened risk for insomnia is crucial for preventing the onset and exacerbation of sleep-related problems. Our findings underscore the importance of sleep reactivity in predicting insomnia severity in this population. Future prospective studies, incorporating objective sleep-related measures, are needed to further investigate the role of sleep reactivity, as well as to explore potential interventions to mitigate insomnia in adults with ADHD.

## Data Availability

The raw data supporting the conclusions of this article will be made available by the authors, without undue reservation.
